# Phosphorylation of Vasodilator-Stimulated Phosphoprotein (VASP) Dampens Hepatic Ischemia-Reperfusion Injury

**DOI:** 10.1371/journal.pone.0029494

**Published:** 2011-12-22

**Authors:** David Köhler, Philipp Birk, Klemens König, Andreas Straub, Therese Eldh, Julio C. Morote-Garcia, Peter Rosenberger

**Affiliations:** 1 Department of Anesthesiology and Intensive Care Medicine, University Hospital, Tübingen, Germany; 2 Clinic of Anesthesiology, Intensive Care Medicine and Pain Therapy, University Hospital Frankfurt am Main, Johann Wolfgang Goethe University, Frankfurt, Germany; 3 Department of Radiation Oncology, University Hospital, Tübingen, Germany; University of Colorado Denver, United States of America

## Abstract

Recent work has demonstrated that the formation of platelet neutrophil complexes (PNCs) affects inflammatory tissue injury. Vasodilator-stimulated phosphoprotein (VASP) is crucially involved into the control of PNC formation and myocardial reperfusion injury. Given the clinical importance of hepatic IR injury we pursued the role of VASP during hepatic ischemia followed by reperfusion. We report here that *VASP* ^−/−^ animals demonstrate reduced hepatic IR injury compared to wildtype (WT) controls. This correlated with serum levels of lactate dehydrogenase (LDH), aspartate (AST) and alanine (ALT) aminotransferase and the presence of PNCs within ischemic hepatic tissue and could be confirmed using repression of VASP through siRNA. In studies employing bone marrow chimeric mice we identified hematopoietic VASP to be of crucial importance for the extent of hepatic injury. Phosphorylation of VASP on Ser^153^ through Prostaglandin E1 or on Ser^235^ through atrial natriuretic peptide resulted in a significant reduction of hepatic IR injury. This was associated with a reduced presence of PNCs in ischemic hepatic tissue. Taken together, these studies identified VASP and VASP phosphorylation as crucial target for future hepatoprotective strategies.

## Introduction

Surgical procedures such as liver transplantation, partial hepatic resection, hepatic tumor surgery or trauma repair may induce hepatic ischemia-reperfusion (IR) injury which is associated with clinically significant reduced liver function [1]. A pathophysiological consequence of the cessation of blood supply followed by reperfusion is cellular damage within the ischemic areas. Several underlying mechanisms are involved in this process such as microvascular dysfunction and enhanced leukocyte-endothelial cell adhesion during the early stages of reperfusion [2]. In addition, triggered by inflammatory mediators (e.g. cytokines) neutrophils are activated to cross the endothelial barrier and translocate into hepatic tissue. This results in the release of enzymes and reactive oxygen species that aggravate the associated tissue injury within the hepatic tissue [3], [4], [5].

Recent work has described an important role for platelet-neutrophil complexes (PNCs) during inflammatory and ischemic tissue injury. In a study by Zarbock et al. the authors demonstrate that the degree of lung injury can be significantly attenuated if the formation of PNCs is blocked [6]. Furthermore, through platelet depletion or the injection of activated platelets the role of platelet-neutrophil complexes (PNCs) that are primed for adhesion, migration, phagocytosis and intracellular killing was further delineated in the past [7], [8], [9]. In a recent study we were able to describe the role of PNCs during myocardial IR injury [10]. In this study we identified vasodilator-stimulated phosphoprotein (VASP), a central cytoskeleton protein affecting actin dynamics, to be a crucial regulator of PNC formation during the reperfusion phase. Phosphorylation of VASP on serine 157 (Ser^157^) through prostaglandin E1 (PGE_1_) or on serine 239 (Ser^239^) through atrial natriuretc peptide (ANP) resulted in a significant attenuation of this PNC formation [11], [12]. Previous work has implicated a hepatoprotective potential of PGE_1_ in cirrhotic patients and in experimental in vivo studies. This hepatoprotective potential was also described for ANP with a reduction of hepatic apoptosis and tissue injury through an ANP infusion during hepatic reperfusion [13], [14], [15], [16].

Given the importance of PNCs for the extent of inflammatory organ injury and the fact that VASP phosphorylation affects the formation of PNCs, we analyzed the role of VASP and VASP phosphorylation during hepatic IR injury. In vivo experimental data depicted a significant reduction of hepatic IR injury in *VASP* ^−/−^ mice associated with a reduced presence of PNCs. Targeted repression of VASP using siRNA confirmed these results. Hepatic IR experiments in bone marrow chimeric animals identified hematopoietic VASP expression to affect the extent of hepatic IR injury. Furthermore, phosphorylation of VASP affects the formation of PNCs and as such the extent of hepatic IR injury.

## Materials and Methods

### Ethic Statement

All animal protocols were in accordance with the German guidelines for use of living animals and were approved by the Institutional Animal Care and Use Committee of the Tübingen University Hospital and the Regierungspräsidium Tübingen. *VASP* ^−/−^ mice were generated, validated and characterized as described previously [17]. The WT controls (C57BL/6 mice) were bred as littermates of *VASP* ^−/−^ mice.

### Murine model of hepatic ischemia

*VASP* ^−/−^ mice and littermate controls were selected to be similar in age-, gender- and weight. After anesthesia was induced animals were placed on a temperature-controlled and heated table to maintain body temperature at 37°C. Following midline laparotomy the liver was exposed. Ligation of the hepatic artery occured in the ligamentum hepatoduodenale, where the portal triad was completely and reversible occluded. During the ischemic period the lobus dexter and lobus caudatus remained perfused via separate influx and efflux, as described previously [18]. Tissue damage was determined through blood serum levels of lactate dehydrogenase (LDH) (Randox, Crumlin, United Kingdom), aspartate (AST) and alanine aminotransferase (ALT) (Teco Diagnostics, Anaheim, USA).

### Pharmacological compounds used

PGE_1_ (0.42 µg/kg/h, Sigma-Aldrich, Munich, Germany), atrial natriuretic peptide (ANP) (0.12 µg/kg/h, Sigma-Aldrich, Munich, Germany) or vehicle (0.9% NaCl) was administered by intravenous infusion beginning 5 min prior to reperfusion and continuing 1 hour during reperfusion.

### In vivo small interfering RNA Repression

To achieve in vivo repression of VASP we used 2.6 µg/g body weight VASP ON-TARGETplus® SMARTpool Mouse siRNA dissolved in 5% Glucose-solution. As control served non-targeting siRNA (Thermo Scientific Dharmacon, Dreieich, Germany) with at least four mismatches to any human, murine or rat gene. The siRNA target sequences of the VASP pool were, J-046659-17 with target sequence UGC CAU UGC UGG AGC CAA A, J-046659-18 with target sequence AGG AAA UCA UCG AAG UCU U, J-046659-19 with target sequence GGG CUA CUG UGA UGC UUU A, J-046659-20 with target sequence GAG CUG AGG AAG CGG GGU U. SiRNA was administered 24 hours before ischemia or in subset of experiments 5 min before onset of reperfusion.

### Western blots for VASP and VASP phosphorylation

Mice were injected for stimulation with either 0.42 µg PGE_1_ or 0.12 µg ANP and blood was taken after 15 min. After spinning at 14,000×g for 10 min to remove cell debris, the pellet was resuspended in RIPA buffer and protein concentration was measured. For siRNA experiments the knockdown was validated by taking blood from mice after 24 h incubation. Primary antibodies were polyclonal anti-VASP (Cell Signaling), polyclonal anti-pVASP^153^ (Cell Signaling) and anti-pVASP^235^ (Cell Signaling). Loading conditions were controlled by polyclonal anti-GAPDH (Santa Cruz Biotechnology).

### Immunohistochemistry of neutrophils and platelets in murine tissue

Immunohistochemical staining was performed with Vectastain® ABC Kit (Linaris, Wertheim, Germany). After inhibiting the non-specific binding sites with avidin blocking solution (Vector Laboratories, CA, USA) the sections were incubated with primary antibody (polyclonal rabbit anti-mouse CD41, Abcam) over night at 4°C. Tissue sections were then incubated with biotinylated anti rabbit IgG for 1 hour followed by Vectastain® ABC Reagent for 30 minutes, and then developed using DAB substrate. For neutrophil staining, the procedure was repeated using rat anti-mouse neutrophil antibody (rat antimouse Ly-6B2 clone 7/4, AbD Serotec) and histogreen as substrate (Linaris). Counterstaining was performed using nuclear fast red (Linaris).

### Triphenyltetrazolium chloride staining

After 30 min ischemia and 3 h reperfusion parts of the lobus medianus were excised washed in ice-cold 0.9% saline, placed on parafilm, frozen at −20°C for 30 min and cut into 1 mm slices. The slices were incubated with 1% TTC at 37°C for 30 min and fixed in 10% formalin. As previously described TTC stains all cells red except those that are depleted in NADPH and therefore allows to distinguish viable tissue from ischemic tissue [19].

### Generation of bone marrow (BM) chimeras

In short, male donor mice (8–10 weeks old, 20–25 g) were euthanized, marrow harvested by flushing the marrow cavity and bone marrow cells were then centrifuged at 400×g for 5 min, resuspended and counted. Recipient mice (8–10 weeks of age, 20–25 g) were irradiated with a total dose of 10 Gy from a 137Cs source. Immediately after irradiation, 10^7^ BM cells/recipient were injected in 0.15 ml 0.9% sodium chloride into the tail vein. The resulting chimeric mice were housed in microisolators for at least 6 weeks before experimentation and fed with water containing tetracycline (100 mg/l) in the first two weeks following BM transplantation. Bone marrow cells were transplanted to generate (1) [WT → WT] (2) [*VASP* ^−/−^ → *VASP* ^−/−^] mice as controls and (3) [WT → *VASP* ^−/−^] (4) [*VASP* ^−/−^ → WT] chimeric mice [20].

### Flow Cytometry

For flow cytometry blood was gently collected by puncture of the hepatic portal vein and immediately anticoagulated. For the determination of PNCs citrated whole blood samples were incubated with a monoclonal anti-CD42b-FITC antibody (clone Xi1.G5, Emfret Analytics) and with a monoclonal anti-CD15-PE antibody (clone C3D-1 Santa Cruz Biotechnology). For the determination of GPIIb/IIIa activation, citrated whole blood samples were incubated with anti-CD42b-FITC Ab (Emfret) and with the activation-specific monoclonal Jon-A-PE Ab (clone Jon-A, Emfret). After 30 minutes incubation at 37°C, samples were fixed using CellFix (BD Biosciences). Flow cytometry was performed on a FACScan cytometer (BD Biosciences) and PNC formation as well as GPIIb/IIIa activation were analyzed according to previously described principles [21], [22]. In all experiments, suitable isotype Abs were used to adjust for nonspecific Ab binding.

### Data analysis

All values are expressed as mean ± SEM. Using Kolmogorov-Smirnov test we could show that the measured values were approximately normally distributed. Statistical significance was determined using one-way ANOVA followed by Bonferroni's multiple-comparison test. Student's *t* test was used where appropriate. A value of *P*<0.05 was considered significant.

## Results

### Hepatic IR injury and PNC infiltration is attenuated in *VASP* ^−/−^ mice

**Figure 1:**
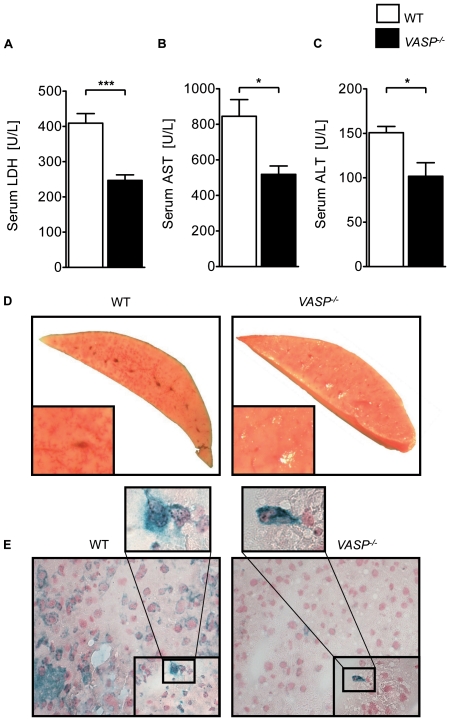
*VASP* ^−/−^ animals demonstrate reduced hepatic IR injury. **A**) LDH-serum levels of *VASP* ^−/−^ and WT mice after 30 minutes ischemia and 3 hours reperfusion in U/L. **B**) Correlating serum levels of AST and **C**) ALT of *VASP* ^−/−^ and WT animals. **D**) Representative TTC stained liversections of *VASP* ^−/−^ and WT animals **E**) Histological images of platelet-neutrophil complexes (neutrophil  =  blue; platelet  =  black) in tissue sections of ischemic tissue of *VASP* ^−/−^ and WT animals (Data are shown as Mean ± SEM, n = 6, *P<0.05; ***P<0.001 as indicated, tissue sections magnification x400 and x1000 with detail sector magnification, n = 3, one representative of 3 individual experiments is demonstrated).

In a model of murine hepatic IR injury we exposed previously characterized *VASP* ^−/−^ and controls to 30 minutes of partial liver ischemia followed by 3 hours of reperfusion [17]. Following this *VASP* ^−/−^ mice demonstrated a significant reduction of LDH, AST and ALT serum levels compared to controls (Figure 1 A, B and C). TTC staining of liver tissue sections confirmed these results (Figure 1 ). Following double staining for PNCs we found a reduced presence of PNCs within tissue of *VASP* ^−/−^ animals compared to controls (Figure 1 E). To determine the presence of PNCs within the blood of these animals we determined the number of PNCs in whole blood samples after 30 minutes ischemia. We found that the number of PNCs was significantly lower in *VASP* ^−/−^ animals compared to WT controls (Figure S1 A). We also found reduced presence of the active state of the murine GPIIb/IIIa receptor on the platelet surface of *VASP* ^−/−^ animals compared to WT controls, thus confirming previously published data from our group (Figure S1 B).

**Figure 2:**
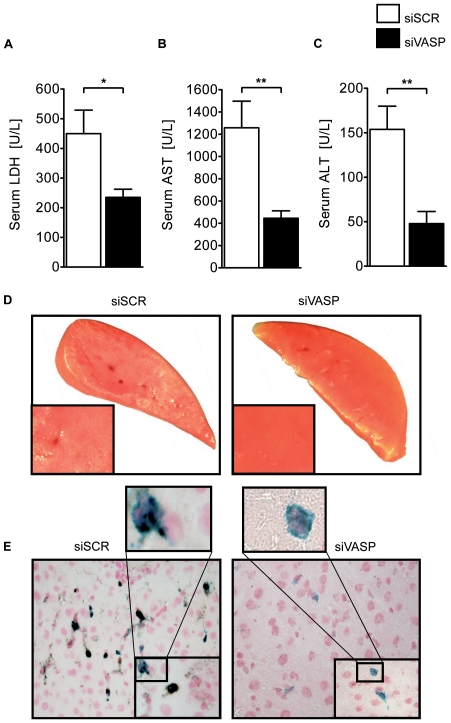
*In-vivo* VASP repression by siRNA dampens hepatic IR injury. **A**) LDH-serum levels after hepatic IR in in-vivo targeted repression of VASP with siRNA (siVASP) or non-targeting siRNA (siSCR) **B**) Correlating serum levels of AST and **C**) ALT of siVASP and siSCR treated WT animals. **D**) Representative TTC stained liversections of both groups. **E**) Histological images of platelet-neutrophil complexes (neutrophil  =  blue; platelet  =  black) in tissue sections of ischemic tissue of siVASP and siSCR treated animals (Data are shown as Mean ± SEM, n = 6, *P<0.05; **P<0.01 as indicated, tissue sections magnification x400 and x1000 with detail sector magnification, n = 3, one representative of 3 individual experiments is demonstrated).

To gain further insight into the specific role of VASP we employed gene targeted repression using siRNA and injected animals 24 hours prior to the start of the experiment with siVASP or non-targeting siRNA (siSCR). Repression of VASP was controlled by Western Blot analysis (Figure S2). We then subjected the siRNA treated animals to the hepatic IR model and found reduced LDH, AST and ALT serum levels in siVASP treated animals compared to siSCR controls (Figure 2 A, B and C). TTC stained liver tissue sections corroborated the reduced tissue damage in the siVASP group (Figure 2 D). This reduction of decreased hepatic damage was associated with a reduced presence of PNCs within the affected hepatic tissue (Figure 2 E). Using siVASP immediately during the reperfusion phase however failed to show a clear effect on IR injury (Figure S6 A, B, C, D and E).

### Hematopoietic VASP repression dampens hepatic IR injury and PNC infilatration

**Figure 3:**
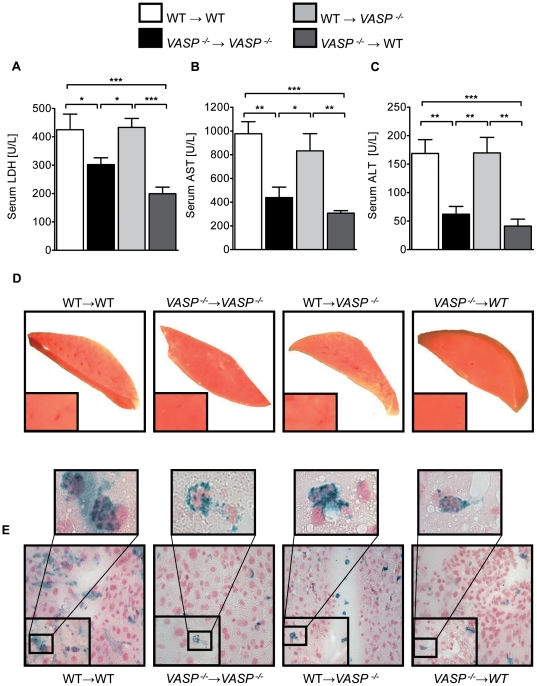
Hematopoietic VASP repression reduces hepatic IR injury. **A**) LDH-serum levels in chimeric animals (*VASP* ^−/−^→WT and WT→*VASP* ^−/−^) and control transplanted animals after 30 minutes of hepatic ischemia followed by 3 hours reperfusion. **B**) Correlating serum levels of AST and **C**) ALT of the chimeric and control animals **D**) Representative TTC stained liversections of ischemic tissue. **E**) Representative histological images of infracted hepatic areas of infiltrated platelet-neutrophil complexes (neutrophil  =  blue; platelets  =  black) of chimeric animals (*VASP* ^−/−^→WT and WT→*VASP* ^−/−^) and control transplanted (WT→WT and *VASP* ^−/−^→ *VASP* ^−/−^) animals (Data are shown as Mean ± SEM, n = 6, *P<0.05; **P<0.01; ***P<0.001 as indicated, tissue sections magnification x400 and x1000 with detail sector magnification, n = 3, one representative of 3 individual experiments is demonstrated).

To gain further insight into the underlying mechanism, we generated chimeric animals to identify whether tissue specific or hematopoietic expression would be involved in the previously observed results. For this, we transferred hematopoietic cells of *VASP* ^−/−^ mice into WT animals (*VASP* ^−/−^→WT) and WT hematopoietic cells in *VASP* ^−/−^ mice (WT→*VASP* ^−/−^), with WT→WT and *VASP* ^−/−^→*VASP* ^−/−^ transplanted mice as controls. To control successful transfer of bone marrow we used western blot analysis (Figure S3). The generated bone marrow chimeric animals were then subjected to 30 minutes ischemia followed by 3 hours reperfusion. Comparison of the serum parameters LDH, AST and ALT in bone marrow chimeric mice demonstrated reduced damage in the hematopoietic *VASP* ^−/−^ (*VASP* ^−/−^→WT; *VASP* ^−/−^→*VASP* ^−/−^) animals (Figure 3 A, B and C). TTC stained liver tissue slices of the different animal groups confirmed the serum measurements (Figure 3 D). Additionally we found a reduced presence of PNCs within the hepatic tissue of hematopoietic *VASP* ^−/−^ mice compared to hematopoietic WT animals. This correlated with decreased hepatic tissue injury (Figure 3 E). With these findings we were able to confirm the findings of a previous study demonstrating the role of VASP during myocardial ischemia-reperfusion [10]. In this study we found that VASP within platelets is crucially important for the observed protective role of VASP repression during IR injury.

### Phosphorylation of VASP on Ser^153^ using PGE_1_ dampens hepatic IR injury

**Figure 4:**
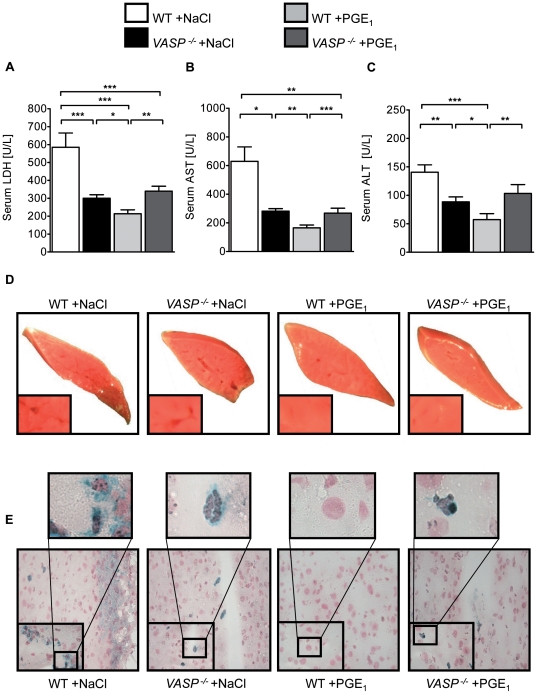
VASP phosphorylation on Ser^153^ using PGE_1_ dampens hepatic IR injury. **A**) LDH-serum levels in WT and *VASP* ^−/−^ animals after 30 minutes of hepatic ischemia followed by 3 hours reperfusion with infusion of saline (vehicle) or PGE1 during reperfusion. **B**) Correlating serum levels of AST and **C**) ALT of vehicle or PGE1 treated WT and *VASP* ^−/−^ animals. **D**) Representative TTC stained liversections of ischemic tissue. **E**) Histological images of platelet-neutrophil complexes (neutrophil  =  blue; platelet  =  black) in tissue sections of ischemic liver lobes of vehicle or PGE_1_ treated WT and *VASP* ^−/−^ animals (Data are shown as Mean ± SEM, n = 6, *P<0.05; **P<0.01; ***P<0.001 as indicated, tissue sections magnification x400 and x1000 with detail sector magnification, n = 3, one representative of 3 individual experiments is demonstrated).

PGE_1_ induces solid human VASP phosphorylation on Ser^157^. In an initial experiment we evaluated possible hemodynamic changes following perfusion with PGE_1_ but did not find a significant change of hemodynamic parameters, however we found the expected murine VASP Ser^153^ phosphorylation. We also observed a cross phosphorylation on VASP Ser^235^ through PGE_1_ (Figure S4 and S5). The PGE_1_ infusion resulted in a significant reduction in LDH, AST and ALT (Figure 4 A, B and C) in WT mice, which was not present in *VASP* ^−/−^ animals. TTC staining confirmed the results of the serum enzyme levels demonstrating reduced hepatic IR injury (Figure 4 D). We then proceeded to identify the presence of PNCs within hepatic tissue of these animals, and found that WT animals treated with PGE_1_ had a minimal number of PNCs in comparison to vehicle treated and PGE_1_ treated *VASP* ^−/−^ mice (Figure 4 E).

### Phosphorylation of VASP on Ser^235^ using ANP attenuates hepatic IR injury

**Figure 5:**
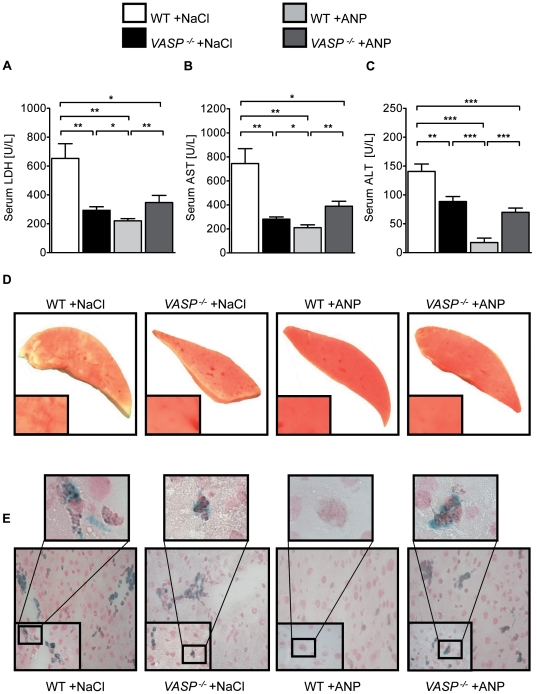
VASP phosphorylation on Ser^235^ using ANP dampens hepatic IR injury. **A**) LDH-serum levels in WT and *VASP* ^−/−^ animals after 30 minutes of hepatic ischemia followed by 3 hours reperfusion with infusion of saline (vehicle) or ANP during reperfusion. **B**) Correlating serum levels of AST and **C**) ALT of vehicle or ANP treated WT and *VASP* ^−/−^ animals. **D**) Representative TTC stained liversections of ischemic tissue. **E**) Histological images of platelet-neutrophil complexes (neutrophil  =  blue; platelet  =  black) in tissue sections of ischemic liver lobes of vehicle or ANP treated WT and *VASP* ^−/−^ animals (Data are shown as Mean ± SEM, n = 6, *P<0.05; **P<0.01; ***P<0.001 as indicated, tissue sections magnification x400 and x1000 with detail sector magnification, n = 3, one representative of 3 individual experiments is demonstrated).

Atrial natriuretic peptide induces human VASP Ser^239^ phosphorylation in a cGMP dependent fashion [23]. To evaluate whether this phosphorylation site also holds protective potential we initially tested for the expected phosphorylation of murine VASP at Ser^235^ (Figure S5). To preclude possible hemodynamic changes following ANP perfusion we determined the blood pressure values in ANP treated animals but did not find a significant change of hemodynamic parameters (Figure S4). When using ANP to induce VASP Ser^235^ phosphorylation in the reperfusion period we also found a significant reduction of serum LDH, AST and ALT levels in WT mice. This was not observed in the *VASP* ^−/−^ mice (Figure 5 A, B and C). TTC staining of liver sections corroborated our findings (Figure 5 D) and correlated with a reduced number of PNCs within the hepatic tissue of WT animals treated with ANP compared to vehicle treated and *VASP* ^−/−^ mice (Figure 5 E).

## Discussion

We have previously demonstrated that the formation of PNCs can be influenced through the expression and phosphorylation of VASP during ischemia-reperfusion of the myocardium [10]. However, the role of VASP during hepatic IR injury remained unclear to date. As an extension of our previous work we demonstrate here that hepatic IR injury is significantly influenced through VASP expression and VASP phosphorylation. This finding was associated with a reduced presence of PNCs within hepatic tissue of *VASP* ^−/−^ mice and associated with reduced hepatic injury following reperfusion.

Tissue injury during an acute inflammatory process or ischemia is significantly affected by the formation of PNCs. Previous work by Zarbock et al. has recognized the role of PNCs during acute lung injury and an inhibition of PNC formation results in a marked decrease of pulmonary injury and improves pulmonary function [6]. Weissmüller et al. demonstrated that PNCs are translocated across the intestinal barrier and that the translocated platelets are functionally sound to release their contents [24], [25]. In a recent study of our group, we were able to demonstrate that PNCs are translocated into ischemic myocardium following reperfusion [10]. In this study we found that the formation of PNCs was significantly dependent on the phosphorylation of VASP. The phosphorylation of VASP achieved through ANP or PGE_1_ resulted in a reduction of GP IIb/IIIa activation on the surface of platelets and in a reduced formation of PNCs between platelets and neutrophils. As a result, the number of circulating PNCs is significantly attenuated and a reduced number of PNCs is translocated into ischemic tissue to aggravate the reperfusion injury. Previous work has shown that the extent of hepatic IR injury is also determined by the activation status of platelets. Cywes et al. demonstrated that an inactivation of platelets with formaldehyde significantly reduces the extent of hepatic IR injury. Furthermore, an activation of platelets resulted in an increased hepatic IR injury [26]. A crucial determinant of the adherence of platelets on the endothelial surface is P-selectin. Animals deficient of P-selectin demonstrate reduced hepatic reperfusion injury following ischemia [27]. Furthermore, the adhesion of platelets to the hepatic endothelium through fibrinogen deposition on endothelial ICAM-1 aggravates hepatic IR injury [28]. Fibrinogen binding to platelets is mediated by the GP IIb/IIIa receptor, yet the surface expression of this receptor is significantly affected by the phosphorylation of VASP [10]. Yadav et al. also demonstrated that neutrophils are important contributors to hepatic IR injury and that the recruitment to the hepatic tissue is dependent on P-selectin [29]. In addition, the inactivation of neutrophils through a MAC-1 antibody results in a significant reduction of hepatic IR injury, this further supports the fact that an interaction of neutrophils with platelets is an important determinant of IR injury within the liver and our results are in accordance with these previous studies [30].

In the presented study, we used ANP to selectively induce phosphorylation of VASP on Ser^235^ and PGE_1_ to induce VASP phosphorylation on Ser^153^ [23], [31]. The protective role of a pharmacological intervention with PGE_1_ or ANP during hepatic IR injury was demonstrated by several investigators previously. Hafez et al. were able to show that an infusion of PGE_1_ resulted in reduced expression of endothelial adhesion molecules such as ICAM within the liver and that this intervention resulted in a reduction of the associated hepatic IR injury [14]. This is in line with previous findings demonstrating a reduction of IR injury and an attenuation of intrahepatic inflammation through PGE_1_ [32], [33]. A protective role of an intervention with ANP during hepatic IR injury was initially described by Bilzer et al. [34]. The positive effect of ANP on intrahepatic inflammation and tissue injury was also confirmed by several other investigators [35], [36]. Our results are in accordance with these previous findings demonstrating a protective role for PGE_1_ and ANP during hepatic IR injury. PGE_1_ and ANP phosphorylate VASP in platelets and as such inactivate these platelets. The phosphorylation of VASP reduces platelet activity and the secretion of their contents. In addition the expression of L-selectin on the surface of endothelial cells is attenuated through a phosphorylation of VASP, which reduces the attachment of inflammatory cells to the endothelial surface. In a previous study we were able to demonstrate that tissue specific repression of VASP increases inflammatory tissue injury [20]. Given the fact that we were able to achieve a reduction of hepatic reperfusion injury by approximately 50% demonstrates that tissue specific VASP holds additional protective function, yet this was not the focus of this investigation. Yet we report here for the first time that the observed protective effect of PGE_1_ or ANP during hepatic IR injury is mediated through the phosphorylation of the target protein VASP. This is undermined by the fact that PGE_1_ or ANP were not effective in *VASP* ^−/−^ animals.

In summary, the results of our study are in line with previous investigations demonstrating the importance of PNCs for the extent of inflammatory tissue injury and confirm that an intervention with PGE_1_ or ANP might be tissue protective during hepatic IR injury. VASP phosphorylation on Ser^153^ or Ser^235^ in mice resulted in reduced formation of PNCs and as a result the extent of hepatic IR injury. This work therefore furthers the understanding of the role of VASP during hepatic IR injury and might result in novel therapeutic strategies in the future.

## Supporting Information

Figure S1 **VASP–deficient animals demonstrate reduced PNC formation and attenuated activation of GP IIb/IIIa receptor during hepatic IR injury.** **A**) *VASP* ^−/−^ and WT mice were subjected to 30 minutes hepatic ischemia. Number of PNCs was determined using anti-CD42b-FITC Ab and anti-CD15-PE Ab after ischemia. **B**) Activation status of the murine GPIIb/IIIa receptor evaluated with the activation-specific Jon-A-PE-Ab during flow cytometry. (Data are shown as Mean±SEM, n = 4; **P<0.01; ***P<0.001 as indicated).
(TIF)

Figure S2 **VASP protein expression in WT animals following siRNA injection.** Westernblot analysis of hepatic tissue in WT animals 24 hours post siVASP or siSCR injection (n = 3).
(TIF)

Figure S3 **VASP protein expression in chimeric animals.** Western blot analysis of chimeric animals following BM transplantation demonstrating VASP expression in hepatic tissue or whole blood of WT→WT (control) transplanted animals, hematopoietic WT into *VASP* ^−/−^ animals (WT→*VASP* ^−/−^), hematopoietic *VASP* ^−/−^ in WT animals (*VASP* ^−/−^→WT) and *VASP* ^−/−^→*VASP* ^−/−^ transplanted control animals (Pooled samples of n = 4/ group).
(TIF)

Figure S4 **Hemodynamic values determined during the experimental protocol.** Animals were cannulated with a catheter into the carotid artery and blood pressure measurements determined during anesthesia, ischemia, reperfusion, injection of atrial natriuretic peptide (ANP) or prostanglandin E1 ( PGE_1_) (All Data are Mean ± SEM, n = 6).
(TIF)

Figure S5 **VASP phosphorylation following PGE_1_ or ANP treatment.** Western blots from whole blood samples from WT mice taken 15 minutes following injection with either prostaglandin E1 (PGE_1_) or atrial natriuretic peptide (ANP) controlled for phosphorylation at murine VASP Ser^153^ through PGE1 and at murine VASP Ser^235^ through ANP (Pooled samples of n = 4/ group).
(TIF)

Figure S6 **Liver IR injury in siRNA treated WT animal during reperfusion. A**) LDH-serum levels following hepatic IR in *in-vivo* targeted repression (onset 5 min previous reperfusion) of VASP with siRNA (siVASP) or non-targeting siRNA (siSCR) **B**) Correlating serum levels of AST and **C**) ALT of siVASP and siSCR treated WT animals. **D**) Representative TTC stained images liver sections of both groups. **E)** ) Histological images of platelet-neutrophil complexes (neutrophil  =  blue; platelet  =  black) in tissue sections of ischemic liver lobes of siVASP and siSCR treated animals (Data are shown as Mean ± SEM, n = 6, *P<0.05 as indicated, tissue sections magnification x400 and x1000 with detail sector magnification, n = 3, one representative of 3 individual experiments is demonstrated).
(TIF)
