# Supplementary figures and images for: Phosphorylation of Vasodilator-Stimulated Phosphoprotein (VASP) Dampens Hepatic Ischemia-Reperfusion Injury
Source: PLoS One. 2011 Dec 22;6(12):e29494. doi: 10.1371/journal.pone.0029494 (PMC3245274; doi:10.1371/journal.pone.0029494)

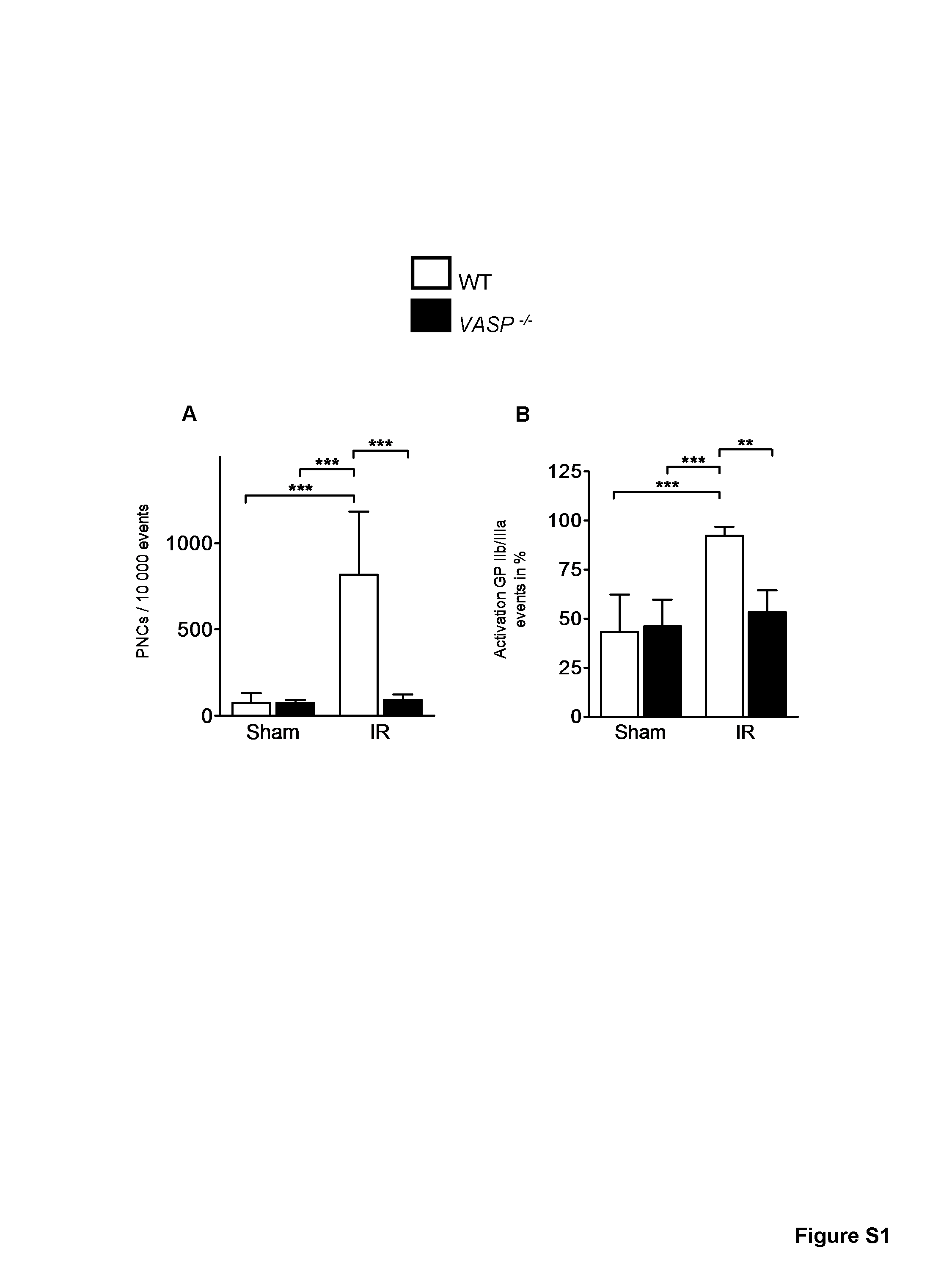

Supplement: Figure S1 — VASP–deficient animals demonstrate reduced PNC formation and attenuated activation of GP IIb/IIIa receptor during hepatic IR injury. A) VASP −/− and WT mice were subjected to 30 minutes hepatic ischemia. Number of PNCs was determined using anti-CD42b-FITC Ab and anti-CD15-PE Ab after ischemia. B) Activation status of the murine GPIIb/IIIa receptor evaluated with the activation-specific Jon-A-PE-Ab during flow cytometry. (Data are shown as Mean±SEM, n = 4; **P<0.01; ***P<0.001 as indicated). (TIF) [file pone.0029494.s001.tif]

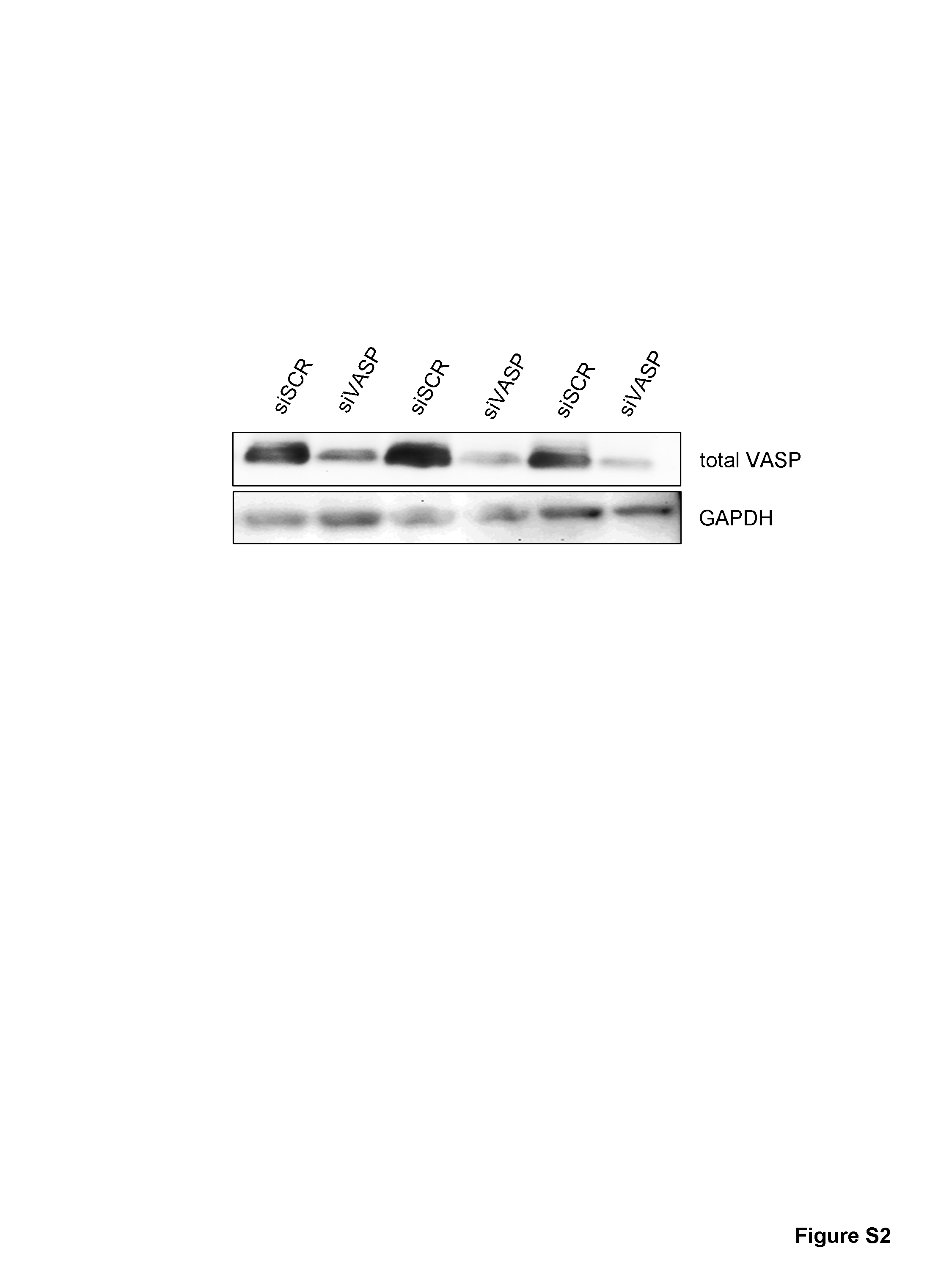

Supplement: Figure S2 — VASP protein expression in WT animals following siRNA injection. Westernblot analysis of hepatic tissue in WT animals 24 hours post siVASP or siSCR injection (n = 3). (TIF) [file pone.0029494.s002.tif]

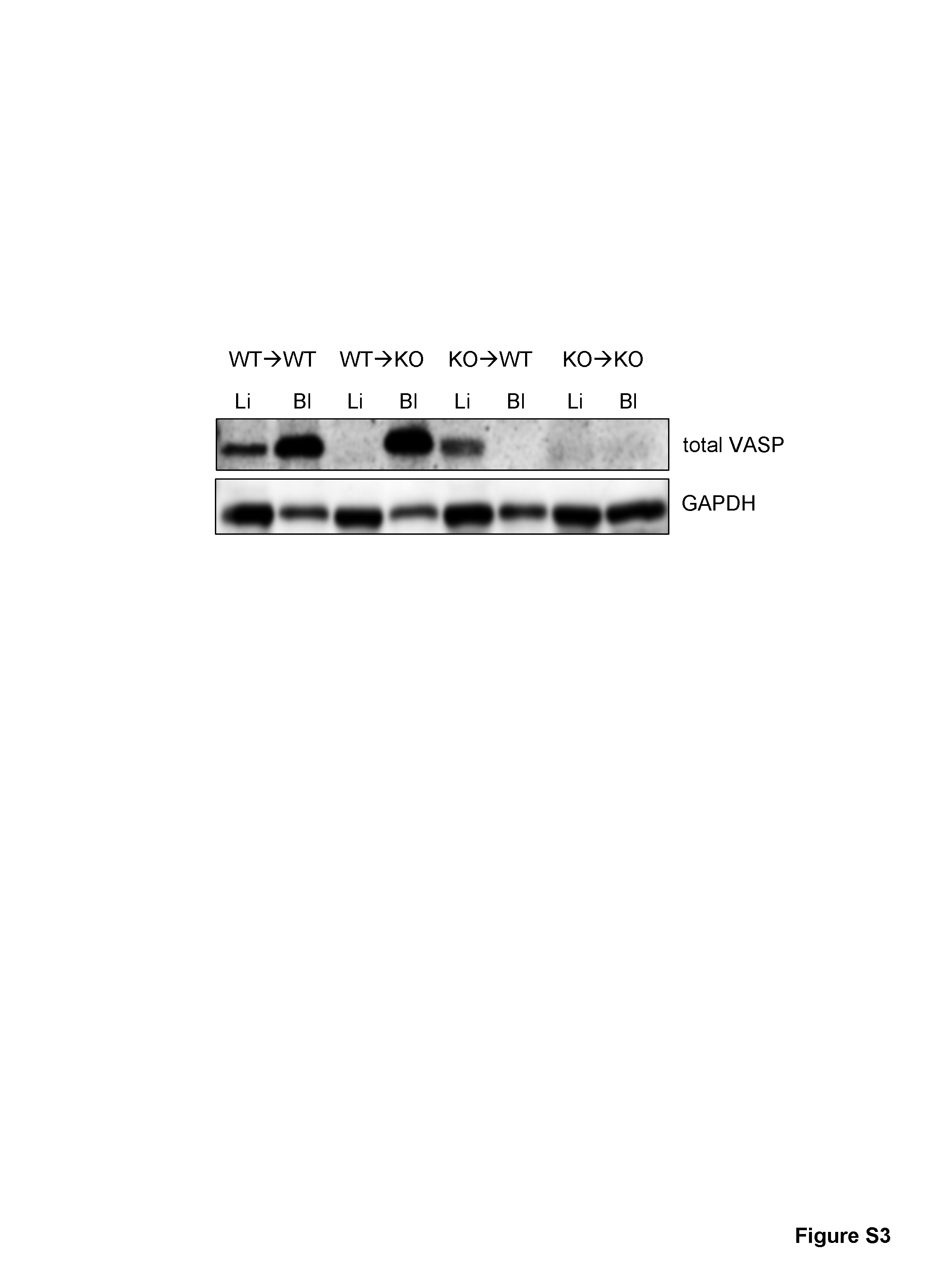

Supplement: Figure S3 — VASP protein expression in chimeric animals. Western blot analysis of chimeric animals following BM transplantation demonstrating VASP expression in hepatic tissue or whole blood of WT→WT (control) transplanted animals, hematopoietic WT into VASP −/− animals (WT→VASP −/−), hematopoietic VASP −/− in WT animals (VASP −/−→WT) and VASP −/−→VASP −/− transplanted control animals (Pooled samples of n = 4/ group). (TIF) [file pone.0029494.s003.tif]

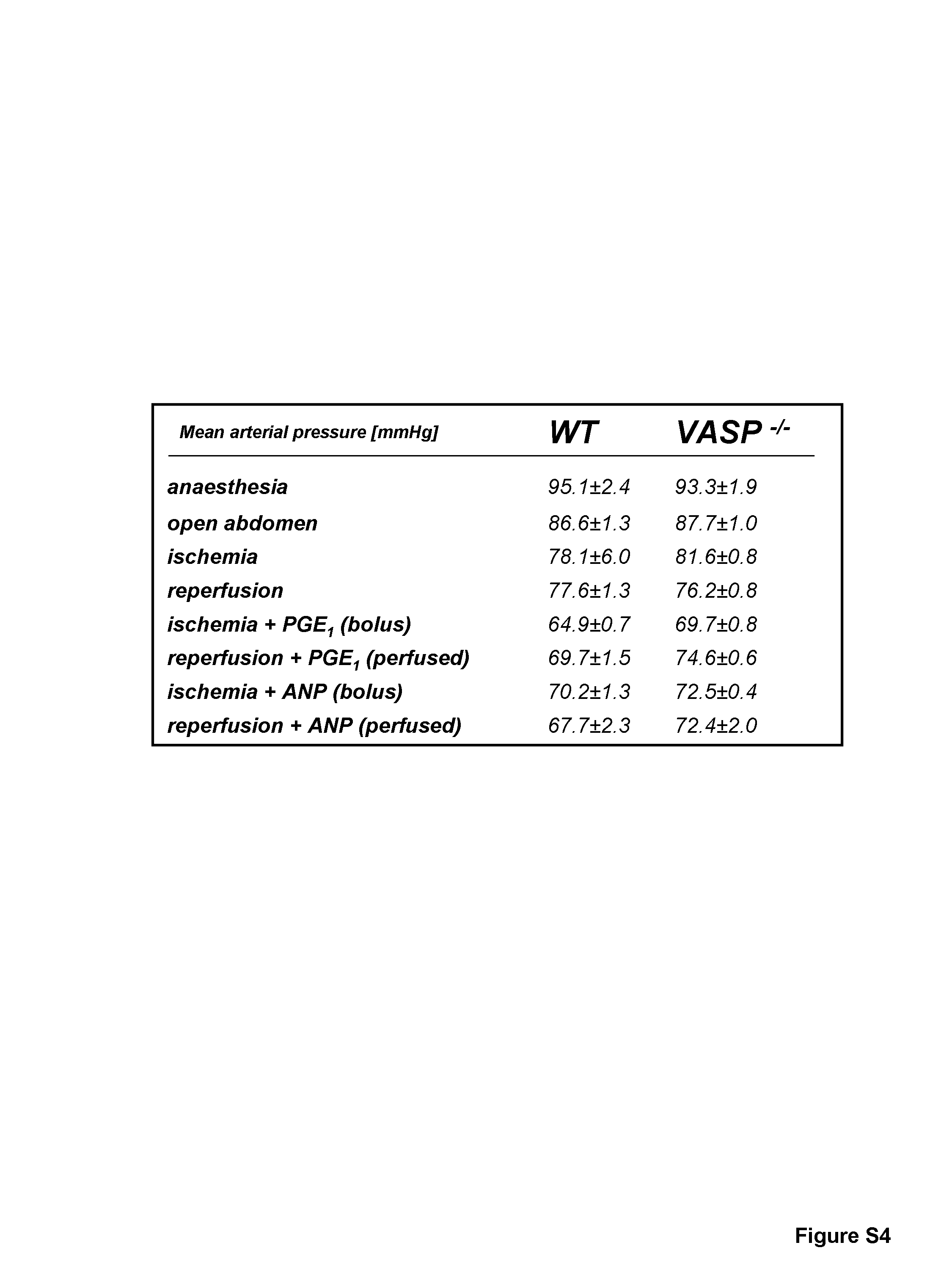

Supplement: Figure S4 — Hemodynamic values determined during the experimental protocol. Animals were cannulated with a catheter into the carotid artery and blood pressure measurements determined during anesthesia, ischemia, reperfusion, injection of atrial natriuretic peptide (ANP) or prostanglandin E1 ( PGE1) (All Data are Mean ± SEM, n = 6). (TIF) [file pone.0029494.s004.tif]

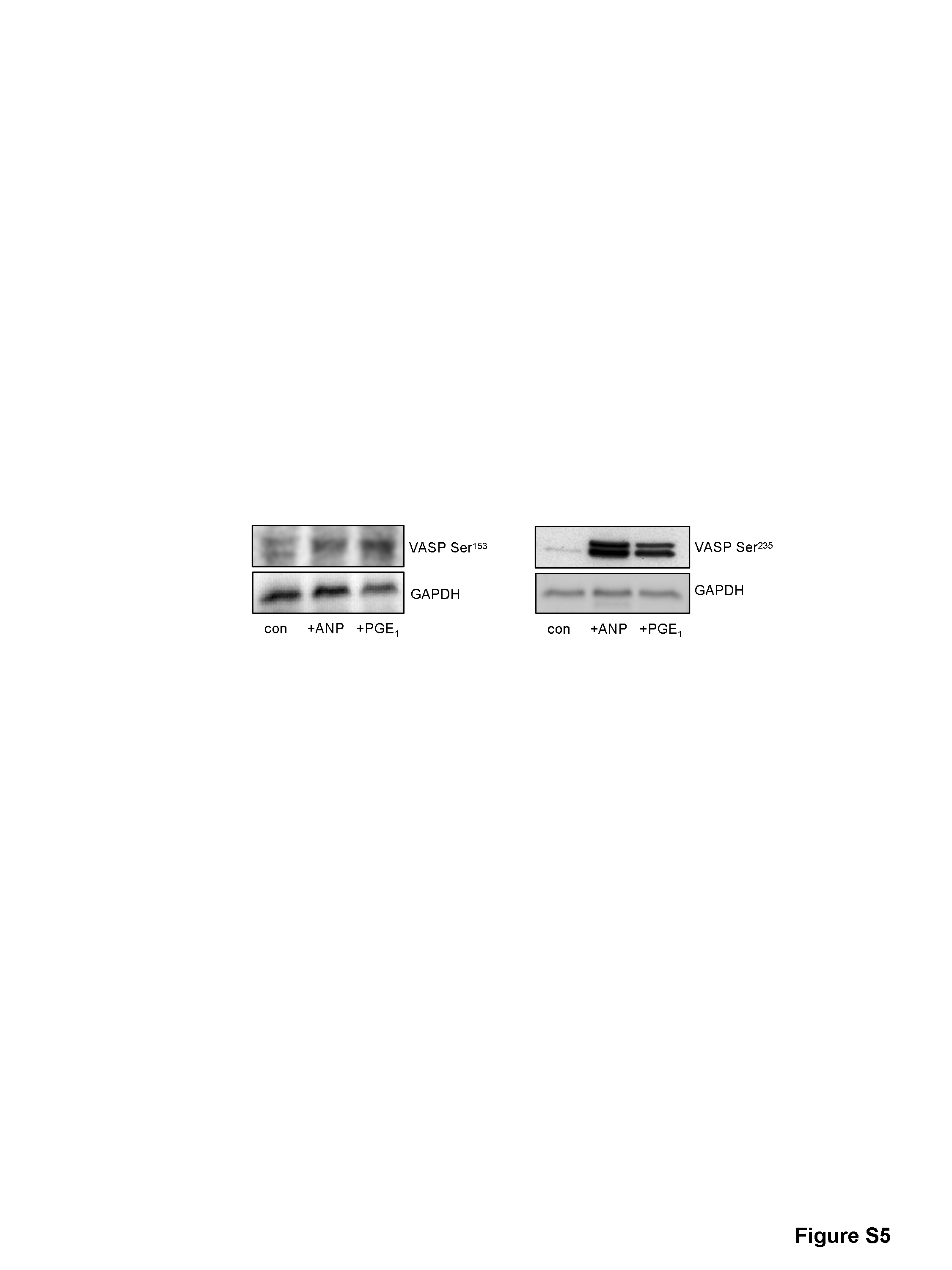

Supplement: Figure S5 — VASP phosphorylation following PGE1 or ANP treatment. Western blots from whole blood samples from WT mice taken 15 minutes following injection with either prostaglandin E1 (PGE1) or atrial natriuretic peptide (ANP) controlled for phosphorylation at murine VASP Ser153 through PGE1 and at murine VASP Ser235 through ANP (Pooled samples of n = 4/ group). (TIF) [file pone.0029494.s005.tif]

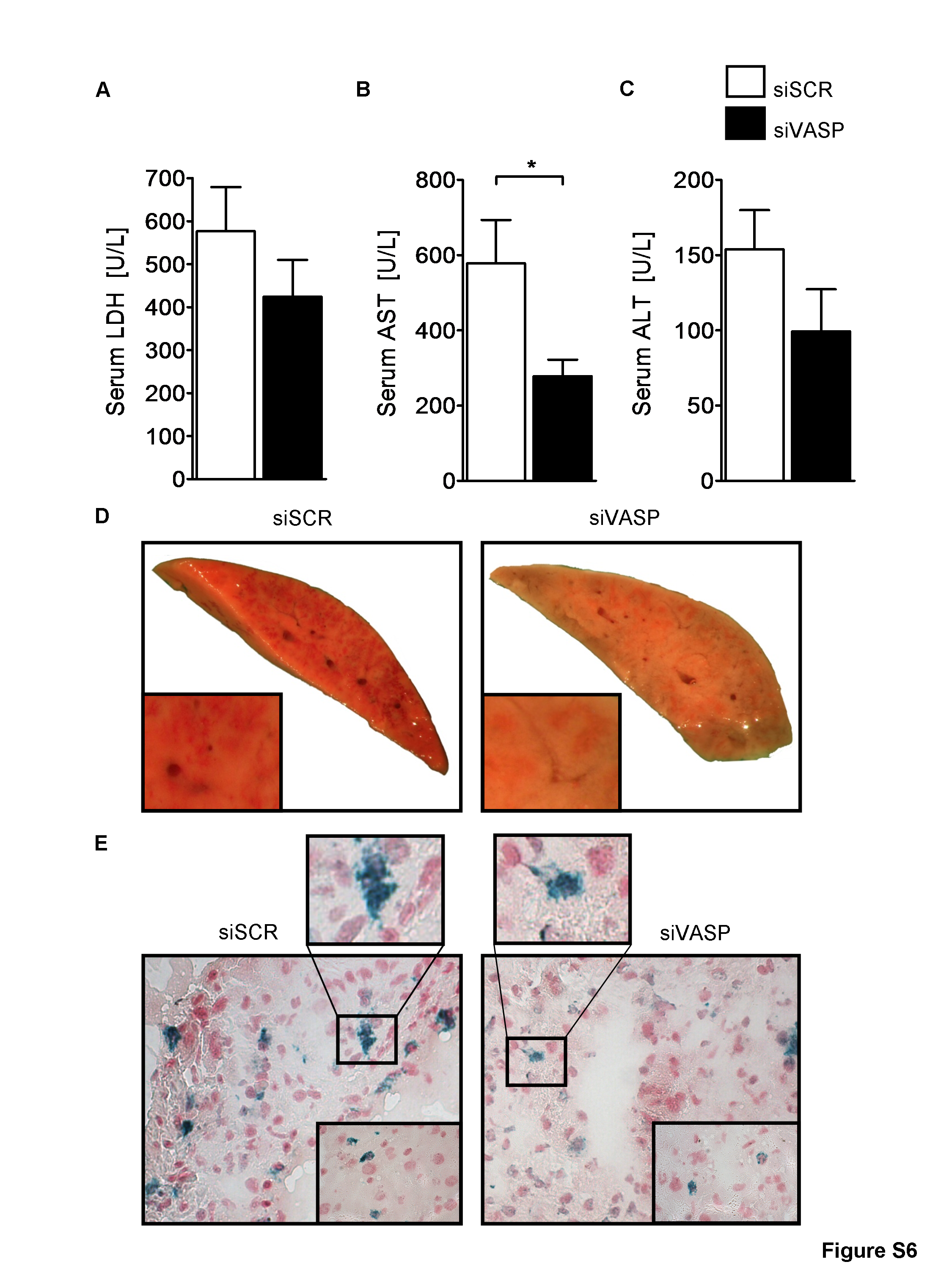

Supplement: Figure S6 — Liver IR injury in siRNA treated WT animal during reperfusion. A) LDH-serum levels following hepatic IR in in-vivo targeted repression (onset 5 min previous reperfusion) of VASP with siRNA (siVASP) or non-targeting siRNA (siSCR) B) Correlating serum levels of AST and C) ALT of siVASP and siSCR treated WT animals. D) Representative TTC stained images liver sections of both groups. E) ) Histological images of platelet-neutrophil complexes (neutrophil = blue; platelet = black) in tissue sections of ischemic liver lobes of siVASP and siSCR treated animals (Data are shown as Mean ± SEM, n = 6, *P<0.05 as indicated, tissue sections magnification x400 and x1000 with detail sector magnification, n = 3, one representative of 3 individual experiments is demonstrated). (TIF) [file pone.0029494.s006.tif]
